# Developmental Outcomes at Ages 3–5 among Very-low and Extremely-low-birthweight Children without Major Complications Assessed at 18–24 Months

**DOI:** 10.24546/0100497750

**Published:** 2025-10-03

**Authors:** NORIKO YAMAOKA, SATOSHI TAKADA

**Affiliations:** 1 Faculty of Nursing, Kobe Tokiwa University, Kobe, Japan; 2 Kobe City Pediatric and General Rehabilitation Center for the Challenged, Kobe, Japan

**Keywords:** Child Behavior Checklist (CBCL) 1.5-5, Developmental outcomes, Japan, Modified Checklist for Autism in Toddlers (M-CHAT), Very-low-birthweight infants

## Abstract

Around 9.4% of infants born in Japan have a low birthweight. While some studies have clarified the developmental progress of very-low-birthweight (VLBW) and extremely-low-birthweight (ELBW) infants at 18 months, less is known about how these children fare in preschool. This study aimed to identify VLBW and ELBW infants without major complications and their behavioral and emotional characteristics as preschoolers compared to their infantile development. Participants were the parents of 28 VLBW and ELBW children without major complications. The Child Behavior Checklist (CBCL) 1.5–5 was administered at ages 3–5, and the results were compared with the Modified Checklist for Autism in Toddlers (M-CHAT). The examination outcomes were assessed at 18–24 months. Of these, 11 had normal scores on all CBCL scales, but the outcomes of the other 17 children were borderline or in the clinical range on either of the CBCL scales. Twenty-one children were screened negative on M-CHAT. Eleven had normal scores on all CBCL scales, but the outcomes of the other 10 children were borderline or in the clinical range on either of the CBCL scales. Seven children screened positive on M-CHAT, and all scored in the borderline or clinical range on either of the CBCL scales. Seven of the 23 M-CHAT items demonstrated relevance between 6 out of 10 CBCL-1.5–5 scales. M-CHAT-positive infants are likely to continue facing behavioral and emotional problems as preschoolers. Even in infants with a negative M-CHAT screening, the underlying problems may become apparent during the preschool years.

## INTRODUCTION

Low birthweight newborns account for 9.4% of all births in Japan, of which 0.4% have a birthweight of 1,000 to 1,500 g and 0.3% have an extremely low birthweight (ELBW: <1,000 g) (1). Improved survival rates for very-low-birthweight (VLBW: <1,500 g) and/or very preterm (<32 weeks of gestation) infants have generated concerns about subsequent neurological and behavioral development. Since these patients often survive complications such as intraventricular hemorrhage and periventricular leukomalacia, most are followed up with throughout a certain period by advanced medical care facilities. Numerous studies have targeted them to identify their problems and solutions and have reported that VLBW and ELBW and/or very preterm infants have an increased risk of long-term sequelae, such as neurodevelopmental disorders, social communication, and behavioral impairments (2, 3). Some VLBW and ELBW infants without major complications are monitored by advanced medical specialists for a relatively shorter period compared to high-risk VLBW infants. Studies in a multicenter cohort in Japan have described the difficulty of continuing follow-ups with VLBW and ELBW infants without major complications (4, 5). This might reflect the fact that only a small number of studies have focused on the prognosis of VLBW and ELBW children without major complications.

In many cases, community healthcare facilities and services provide follow-up care for VLBW and ELBW infants without major complications. In Japan, health checkups are required by the Maternal and Child Health Law. They are conducted for children aged between 18 months and 3 years with the aim of early detection and intervention for neurodevelopmental difficulties. However, despite being assessed as normal at 18-month checkups, underlying behavioral and emotional problems appear in some children at 3 years of age or later. Gargus et al. assessed 3,150 ELBW infants at 18 to 22 months of corrected age who were discharged alive from neonatal intensive care units, and 27% were without impairment (6). Although few studies have focused on the prognosis of VLBW and ELBW and/or very preterm children, studies targeted at moderate and/or late preterm children are increasing, and the results have provided insightful information. These studies indicate that extremely, moderately, and/or late preterm infants could develop behavioral and/or emotional problems in their preschool years or later (7, 8). Nevertheless, the developmental progress of VLBW and ELBW infants without major complications is currently not well defined.

An earlier study acknowledged the significance of the information that predicts preschool-age outcomes of VLBW and ELBW infants without major complications (9). Reliable indicators of adverse outcomes in infancy would be helpful for appropriate detection and intervention to overcome the future occurrence of developmental difficulties (10). This study aimed to identify the behavioral and emotional characteristics of VLBW and ELBW preschoolers without major complications compared to the characteristics of their infantile development. We aimed to determine whether VLBW and ELBW infants without major complications assessed as normal had potential neurodevelopmental problems that became apparent during their preschool years. We also sought to identify the limitations of the predictors to items that can be measured by M-CHAT.

## MATERIALS AND METHODS

### Study design and participants

A prospective longitudinal design was used in this study. Participants were recruited from a 2-year support program for VLBW infants and their parents, managed by a community-based child and family support center in Kobe City, Japan. The support program was divided into four classes according to the infants’ age, and approximately 10 participants participated in the classes that are held monthly. Infants were evaluated from their clinical records and excluded from the study if they were diagnosed with congenital or postnatally acquired abnormalities, affected by overt intellectual or cognitive problems, or exhibited neurodevelopmental deficits or behavioral problems in routine clinical examination. Infants with a high risk of neurodevelopmental deficits, such as severe chronic lung disease, necrotizing enterocolitis, neonatal sepsis, periventricular leukomalacia, and intraventricular hemorrhage above grade II, were excluded.

### Procedures

The first data collection occurred between November 2015 and November 2019, when the infants were 18–24 months old (corrected age). We administered the 4-point scale Japanese version of the Modified Checklist for Autism in Toddlers (M-CHAT) to the study participants’ parents (11). Although the original M-CHAT consists of 23 yes/no questions, parents often have difficulty giving definite answers to yes/no questions (12). Wong et al. adapted the M-CHAT questionnaire into a 4-point scale (*never, seldom, often*, and *usually*) with semi-quantification as 0%, <25%, 25% to 50%, and >50%, respectively (12). For each of the 23 items in the M-CHAT, responses of ‘*never*’ or ‘*seldom*’ were classified as ‘no,’ while responses of ‘*often*’ or ‘*usually*’ were classified as ‘yes.’ The M-CHAT (13) was originally designed to identify signs of autism spectrum disorder (ASD) in children aged 18–30 months. It is completed by parents and is designed for use during regular pediatric checkups. Failure on any three items or two out of six critical items (showing interest in others, protodeclarative pointing, bringing objects to parent, imitating, responding to name, and following a point) was defined as a positive screen for the risk of ASD. The Japanese version of the M-CHAT, in conjunction with the routine 18-month health examination, has successfully detected children with ASD (14).

The second assessment was conducted between September 2017 and July 2021, when the infants reached 3–5 years old. The CBCL-1.5–5 questionnaires were mailed to parents who participated in the M-CHAT screening. The completed CBCL-1.5–5 questionnaires were mailed to the researchers. The Child Behavior Checklist 1.5–5 (CBCL-1.5–5) (15) is a questionnaire that measures the frequency of various social, behavioral, and emotional problems and is widely used in childcare support services and research. It is a standardized test normed on national samples of children from the United States of America. It consists of 99 items, with 1 item recording additional problems. The items were converted into seven syndrome scales: a set of problems that tend to occur together (emotionally reactive, anxious/depressed, somatic complaints, withdrawal, sleep problems, attention problems, and aggressive behavior). Additionally, the CBCL-1.5–5 can be scored in terms of two broadband scales (internalizing and externalizing behaviors) based on the grouping of syndromes. Total problem scale scores were also calculated. The CBCL-1.5–5’s empirically derived scales were developed through factor analysis of data from the general pediatric population, and there is evidence to support the validity and reliability of the problem scales of the test (15). Each syndrome scale score has a normalized corresponding *T*-score. Normalized *T*-scores are assigned to the raw scores of a scale according to the percentiles found for the raw scores in a relevant sample. The *T*-scores of the total problem scales and the two broadband scales were classified as the normal range (less than 60), borderline clinical range (60–63), and clinical range (>63). Seven syndrome scales’ *T*-scores are also classified as normal range (less than 65), borderline clinical range (65–69), and clinical range (70 and over).

### Data analyses

The CBCL-1.5–5 scores were analyzed as a continuous outcome in the original score units. Since the findings did not follow a normal distribution, nonparametric tests were used to analyze the study data. Statistical analyses were performed using the chi-square or Fisher’s exact test to evaluate the differences between the groups screened as negative and positive on M-CHAT, and compare the CBCL-1.5–5 evaluation results between the normal and borderline/clinical ranges groups. We used the Mann-Whitney *U* (M-CHAT answers: *never*/*seldom*) and Kruskal-Wallis (M-CHAT answers: *never*, *seldom*, and *often* or *seldom, often*, and *usually*) tests to calculate the association between the M-CHAT items and the CBCL-1.5–5 scales. Differences were considered statistically significant at *P* < 0.05. All data analyses were conducted with SPSS version 23.0 for Windows (Armonk, NY: IBM Corp.).

### Ethical considerations

This study was approved by the Ethics Committee of Kobe University Graduate School of Health Sciences (No. 411 and No. 411-1), Kobe, Japan, following the World Medical Association Declaration of Helsinki. The research purpose, safety of the participants, voluntary participation in this study, and anonymity of the participants were explained orally. Written informed consent was obtained from all parents during the first data collection period. Parents who participated in the second assessment did not need to sign the consent forms, and the return of the questionnaire implied consent. Appropriate measures were taken to manage participant withdrawals from the study.

## RESULTS

### Sample characteristics and M-CHAT screening

Of the 50 parents who participated in the M-CHAT screening, 28 returned the CBCL-1.5–5, indicating a 56.0% response rate. Twenty-two (44.0%) withdrew from the study. Among them, eight questionnaires were returned from the post office due to unknown forwarding address. During the first data collection, 11 of the 50 infants (22.0%) had a positive M-CHAT score. At the end of the CBCL-1.5–5 questionnaire, 21 of the 28 infants (75.0%) screened negative on M-CHAT, and 7 (25.0%) screened positive. Table I shows an overview of the characteristics of the M-CHAT-negative and M-CHAT-positive screened VLBW infants. There were no statistically significant differences between groups regarding age, birth weight, gestational age, maternal age, sex, or birth order.

### Evaluation of CBCL-1.5–5 in children with negative M-CHAT screening

More than 80% of the M-CHAT-negative children were within the normal range CBCL-1.5–5 scales, excluding attention problems (67%). Of the 21 children screened as M-CHAT-negative, 11 (52.4%) were within the normal range in all CBCL-1.5–5 scales, but the outcomes of 10 children (47.6%) were in the borderline or clinical range in either scale. The outcomes of the CBCL-1.5–5 *T-*scores were compared with the results of the M-CHAT (Table II). Of the 21 children screened as M-CHAT-negative, 5 (24%) were within the borderline range, and 2 (9%) were within the clinical range on the attention problems scale. Three children (14%) were within the clinical range on the withdrawn scale and the total problems scale, and three children (14%) were within the borderline range on the anxious/depressed scales.

### Evaluation of CBCL-1.5–5 in children with positive M-CHAT screening

All seven children who screened positive on M-CHAT scored in the borderline or clinical range on either CBCL-1.5–5 scales. Of the seven children who screened positive on M-CHAT, four were within the borderline range on the attention problems scale. Two were within the clinical range for total problems, internalizing problems, externalizing problems, and anxious/depressed problem scales. The outcomes of the CBCL-1.5–5 *T-*scores were compared with the results of the M-CHAT (Table II).

### Median and IQR of CBCL-1.5–5 *T*-scores compared with M-CHAT results

The median and interquartile range (IQR) of CBCL-1.5–5 *T*-scores of the total problem scales, two broadband scales, and seven syndrome scales compared to the results of the M-CHAT are also presented in Table II. No significant differences were found in the *T*-scores between the groups, but most scores of the M-CHAT positive-screen group were higher than those of the negative group, excluding emotionally reactive score. The M-CHAT negative-screen group’s median CBCL-1.5–5 *T*-scores were within the normal range on either scale. In contrast, the median *T*-scores for total, internalizing, and attention problems were in the borderline range for the M-CHAT positive screening group (60, 61, and 65, respectively).

### Comparison of *T*-scores regarding CBCL-1.5–5 normal and borderline/clinical-range children

Table III shows the comparison of *T-*scores with normal or borderline/clinical range in CBCL-1.5–5. Of the 28 participants in the CBCL-1.5–5 assessment, 11 (39.3%) were within the normal range on all scales, and 17 children (60.7%) scored in the borderline or clinical range on either scale. Of those 17 children, 10 (35.7%) were in the borderline range on either scale. The most common borderline-assessed scale was attention problems (nine children), followed by anxious/depressed problems (four children). The outcomes of seven children (25.0%) were in the clinical range on either scale. The most common clinically assessed scale was total problems (five children), followed by internalizing problems and withdrawal (four children each). The median and IQR of *T*-scores of all scales in comparison with the results of the CBCL-1.5–5 are also shown in Table III. Significant differences between the normal and borderline/clinical-range groups were observed in the attention problem scores (<0.020). Most median *T*-scores for all scales in both groups were within the normal range. However, the median *T*-scores for attention problems in the borderline/clinical-range group were within the borderline range. Furthermore, the median *T*-scores for total and internalizing problems were near the borderline range (59) in the borderline/clinical-range group.

### Association between M-CHAT items and *T*-scores of CBCL-1.5–5 scales

The Mann-Whitney *U* and the Kruskal-Wallis tests were performed to identify the M-CHAT items associated with the *T*-scores of the CBCL-1.5–5 scales. Seven out of 23 M-CHAT items showed relevance between 6 out of 10 CBCL-1.5–5 scales (Table IV). Two of the seven M-CHAT items are critical (14: does not respond to the name, and 15: does not look at the toy when pointing to it). The participants who answered “*usually*” or “*often*” to the M-CHAT items 10 (does not look you in the eye), 15, and 20 (have you wondered if the child is deaf) had significantly higher *T*-scores on the somatic complaints scale. As for the sleep problems scale, participants who responded “*usually*” or “*often*” to items 14 and 17 (does not look at things you look at) had significantly higher *T-*scores. The participants who answered “*usually*” or “*often*” to items 14 and 21 (does not understand what people say) had significantly higher *T*-scores on the anxious/depressed scale. Concerning the internalizing problems scale, the participants who responded “*usually*” or “*often*” to item 15 had significantly higher *T*-scores. As for the withdrawn scale, the participants who answered “*usually*” or “*often*” to item 12 (does not smile in response to smile) had significantly higher *T*-scores. The participants who responded “*usually*” or “*often*” to item 17 had significantly higher *T*-scores on the attention problems scale.

## DISCUSSION

We aimed to determine whether VLBW and ELBW infants without major complications assessed as normal had underlying problems that became apparent during their preschool years. Our results showed that nearly half of the infants screened as normal may face behavioral and emotional problems in their preschool years. We also sought to identify the limitations of the predictors to items that can be measured by M-CHAT. On this point, we found that several M-CHAT items were relevant to some CBCL-1.5–5 scales.

### M-CHAT screening

Previous studies have mentioned that parents of impaired children are more likely to return for follow-up evaluation (4, 16). There is a supposition that parents with responses might be concerned about their children. Focusing on Japanese studies, the positive screening rate at the first stage in Kamio’s study was 17% (14). In another Japanese study on VLBW and ELBW infants at 18 months of age, the positive-screen rate was 32.3%, compared with 12.9% in normal birth weight infants (17). Guy et al. reported that 14.5% of moderately and/or late preterm infants screened positive on M-CHAT, and 2.4% screened true positive (18). You et al. indicated that 8.8% of late preterm infants screened true positive on M-CHAT at 2 years of age (19). Since our study was targeted at VLBW and ELBW infants without major complications and did not report true and false positive rates of the M-CHAT screening, a simple comparison is difficult. However, following these studies, our findings suggest that moderately and/or late preterm infants and VLBW and ELBW infants without major complications may show higher rates of positive M-CHAT screening. Researchers concerned with newborns with extremely low gestational age state that children with motor, vision, or hearing impairments are much more likely to screen positive on the M-CHAT (20, 21), and even those without such impairments showed nearly three times higher rate (16%) of positive screen on M-CHAT than expected in unselected populations (20). Guy et al. indicate that moderately and/or late preterm infants, excluding neurosensory impairments, were associated with a 3.7 times increased risk for a true positive screen (18). The rate of positive screening of M-CHAT for low-risk VLBW infants remains unclear. Still, our findings indicate that the results of the screening need to be carefully interpreted, and other clinical information such as medical and perinatal history, developmental history, family and environmental factors, and behavioral observations should be referred to when assessing VLBW and ELBW infants without major complications (18, 21).

### CBCL-1.5–5 evaluation

This study aimed to determine whether infants without major complications VLBW and ELBW who were assessed normally have underlying problems that become apparent in their preschool years. Our second assessment demonstrates that M-CHAT positive-screen, VLBW and ELBW infants without major complications are likely to continue facing behavioral and emotional problems as preschoolers, and similar difficulties appear in preschool ages in almost half of those who were considered negative for M-CHAT. We found that the M-CHAT positive-screen VLBW and ELBW infants without major complications were at greater risk for borderline/clinical CBCL-1.5–5 scores on total problems, internalizing problems, and attention problems. Internalizing problems consists of four syndrome scales: emotionally reactive, anxious/depressed, somatic complaints, and withdrawn, which comprise problems that are mainly within the self (15). They are significantly associated with VLBW and ELBW and/or very preterm birth (2). Our results are consistent with two previous studies targeting low-risk, moderately preterm, and late preterm children, who reported that they were associated with higher levels of internalizing problems (22, 23). Additionally, earlier research on the developmental outcomes of moderate and/or late preterm children indicates that more attention problems exist in these populations (7, 8, 22, 23). As for the M-CHAT negative-screen VLBW and ELBW infants without major complications, the median CBCL-1.5–5 *T*-scores were within the normal range for all the scales. However, we cannot determine whether negatively screened VLBW and ELBW infants without major complications did not have a risk of behavioral or emotional problems for preschoolers. Notably, seven children (33%) were within the borderline or clinical range on the attention problems scale, three children (14%) were within the clinical range on the withdrawn scale, and three children (14%) were within the borderline range on the anxious/depressed scales. Previous studies have investigated the ability of CBCL-1.5–5 to detect ASD. These studies reveal that the withdrawn scale showed significantly high predictive validity for screening ASD at preschool age (24–26). Preterm infants, as well as infants later diagnosed with ASD, show increased attention, anxiety, depression, and withdrawal symptoms (27). Since developmental profiles change as these children mature (7), M-CHAT negative-screen, VLBW and ELBW preschoolers without major complications within the borderline and/or clinical range on either CBCL-1.5–5 scales would require monitoring and appropriate interventional strategies.

### Possible infantile predictors of preschoolers’ neurodevelopmental outcomes

Another aim of this study was to identify the limitations of the predictors to items that can be measured by M-CHAT. Within the parameters of the M-CHAT items, our findings revealed that seven items showed relevance among the six CBCL-1.5–5 scales. Items 12, 15, and 17 were associated with a one-to-one correspondence with withdrawal, internalizing, and attention problems. As discussed earlier, VLBW and ELBW children without major complications are at a greater risk of experiencing these three problems. Our findings showed the possibility that items 12, 15, and 17 of the M-CHAT may predict neurodevelopmental outcomes of VLBW and ELBW preschoolers without major complications. First, the participants who responded “*usually*” or “*often*” to item 15 (does not look at the toy when you point to it) had significantly higher *T*-scores on the internalizing problems scale. Item 15 was one of the critical items closely related to joint attention (12), which is the ability to share attention with another person regarding an object or event of interest, and its absence is one of the earliest signs of ASD (28). One literature review indicates that preterm infants’ joint attention abilities are impaired compared to those of full-term infants (29). Impaired joint attention corresponds to lower social skills, and preterm children demonstrate less ability to assign goals and beliefs to themselves and others (3). These are the conceivable factors of the association between item 15 and internalizing problems. Second, withdrawal is one of the scales included in internalizing problems. Participants who answered “*usually*” or “*often*” to item 12 (does not smile in response to smile) had significantly higher *T*-scores. Withdrawal is commonly observed in preterm children (27). Smiling is the ordinarily observed social behavior in children, but preterm children often display difficulties in social interaction (29). Interaction difficulties with others are critical symptoms of ASD (3, 27). Possible causes of difficulties in VLBW and/or very preterm infants have been studied across various research fields, and their results are still inconsistent. Nevertheless, withdrawal is common in both the preterm and ASD populations (3, 27). Third, participants who responded “*usually*” or “*often*” to item 17 (does not look at things you look at) had significantly higher *T*-scores on the attention problems scale. Item 17 requires intact vision (20); however, preterm infants demonstrate less visual fixation on social images than term infants and sometimes avoid social gaze, which may be a function of impaired attention (3). Previous research reports that children born 30–34 weeks ranked below term children at 3 years of age on a test of sustained attention (7). The risk of attention deficit hyperactivity disorder (ADHD) increases in both preterm infants and infants later diagnosed with ASD (27). Problems with sustained attention become more common in preterm children with age, even in infants without major complications (30). These findings are supported by Robinson et al., who found that individuals born preterm are at an increased risk of ADHD diagnosis in adulthood (31). The association among the remaining four M-CHAT items and three CBCL-1.5–5 scales were not clarified in this study. However, we can say for now that the four items (10: does not look at you in the eye, 14: does not respond to your name, 20: have you wondered if the child is deaf, and 21: does not understand what people say) require intact vision or hearing (20). The importance of vision was mentioned earlier. Auditory development is closely linked to later language skills, focusing on listening and speaking, making it a basic cognitive development (3). Although there are controversial points in previous studies, the noisy environment of the NICU may affect later neurodevelopmental performance (3).

### Suggestions for early intervention

Our findings indicate the necessity of follow-up assessments from the perspective of early intervention. As previously mentioned in the introduction, 18-month and 3-year health checkups are mandated in Japan. However, even if children are assessed as having no apparent concerns at these checkups, VLBW and ELBW children, with or without major complications, should continue to be monitored at least until the beginning of elementary school. We propose the promotion of health checkups during the later stage of early childhood. Fortunately, the Children and Families Agency has launched a policy promoting the implementation of health checkups at age five, accompanied by increased financial support for local governments (32). This initiative aims to identify and address developmental difficulties at an early stage (32). Pediatricians, public health nurses, and other professionals provide support through consultations with parents and by sharing information with nursery schools, kindergartens, and elementary schools.

### Limitations

The present study has several limitations. First, a critical limitation of our study is that the CBCL-1.5–5 questionnaire was not collected from 44% of the participants in the M-CHAT screening despite thorough action to maximize the response rate. The sample size was too small to conduct regression analysis to clarify the M-CHAT items associated with the CBCL-1.5–5 scales. Second, with the small sample size, the generalizability of our study is limited by the restricted data collection facilities. Third, we did not collect the data on participants’ socioeconomic status. Information such as the level of parental education, annual income, and marital status is essential for the study of VLBW children, especially for the low-risk population. Fourth, our study lacked controls of children with a normal weight. Further research is required to clarify the factors associated with the development of VLBW and ELBW children without major complications.

## CONCLUSION

Our study adds to the limited longitudinal research evaluating the developmental outcomes of VLBW and ELBW children without major complications at ages 3–5 assessed at 18–24 months using M-CHAT. We found that VLBW and ELBW infants without major complications had higher rates of positive M-CHAT screening, as with moderate and/or late preterm infants. We showed that nearly half of the M-CHAT negative-screen, VLBW and ELBW infants without major complications may face behavioral and emotional problems such as withdrawal, anxiety/depression, and attention problems in their preschool years. Our findings have some implications for the possibility that items 12, 15, and 17 of the M-CHAT may be the infantile predictors of low-risk VLBW and ELBW preschoolers’ neurodevelopmental outcomes such as internalizing problems, withdrawal, and attention problems. This study highlights the importance of continuous evaluation for VLBW and ELBW infants with and without major complications, as they may have underlying problems that become apparent in their preschool years.

## Figures and Tables

**Table I:** Characteristics of participants by M-CHAT screening result

	M-CHAT screen negative (N = 21)	M-CHAT screen positive (N = 7)	*P*
Corrected age at the time of M-CHAT screening (mean ± SD)	20.7 ± 2.2 months	20.4 ± 2.2 months	0.530^a^
Age at the time of CBCL-1.5–5 screening (mean ± SD)	52.1 ± 7.4 months	55.3 ± 8.1 months	0.657^a^
Birth weight (mean ± SD)	958 ± 311 g	735 ± 378 g	0.358^a^
Very low birth weight: 1,000–1,500 g at birth	9	1	
Extremely low birth weight: <1,000 g at birth	12	6	
Gestational age (mean ± SD)	28.2 ± 3.2 weeks	27.0 ± 2.6 weeks	0.444^a^
Late preterm: 34–36 weeks of gestation	2		
Moderately preterm: 32–33 weeks of gestation	1		
Very preterm: 28–31 weeks of gestation	9	3	
Extremely preterm: <28 weeks of gestation	9	4	
Maternal age at birth (mean ± SD)	35.1 ± 3.4 years	36.9 ± 4.5 years	0.550^a^
Sex (male/female)	10/11	1/6	0.131^b^
Birth order (first born/subsequent born)	10/11	6/1	0.091^b^

a : Chi-square test,

b : Fisher’s exact test.

**Table II:** Outcomes of CBCL-1.5–5 *T*-scores by M-CHAT screening result

*T*-scores	M-CHAT negative-screen (N = 21)			M-CHAT positive-screen (N = 7)		
	All scores normal range (n = 11) Either scale borderline range (n = 6) Either scale clinical range (n = 4)			All scores normal range (n = 0) Either scale borderline range (n = 4) Either scale clinical range (n = 3)		

	Normal range n (%)	Borderline clinical range n (%)	Clinical range n (%)	Normal range n (%)	Borderline clinical range n (%)	Clinical range n (%)
Total Problems	17 (81)	1 (5)	3 (14)	3 (43)	2 (28)	2 (28)
Internalizing problems	18 (86)	1 (5)	2 (9)	3 (43)	2 (28)	2 (28)
Emotionally Reactive	20 (95)	1 (5)		6 (86)		1 (14)
Anxious/Depressed	18 (86)	3 (14)		4 (58)	1 (14)	2 (28)
Somatic Complaints	21 (100)			4 (58)	2 (28)	1 (14)
Withdrawn	18 (86)		3 (14)	5 (72)	1 (14)	1 (14)
Externalizing Problems	18 (86)	2 (9)	1 (5)	4 (58)	1 (14)	2 (28)
Attention Problems	14 (67)	5 (24)	2 (9)	3 (43)	4 (58)	
Aggressive Behavior	21 (100)			5 (72)	1 (14)	1 (14)
Sleep problems	20 (95)	1 (5)		4 (58)	2 (28)	1 (14)

*T-*scores	Median (IQR)	Median (IQR)	*P*-value
Total Problems	48 (45–57)	**60** (53–71)	0.458
Internalizing problems	53 (41–58)	**61** (53–72)	0.183
Emotionally Reactive	51 (50–56)	51 (50–64)	0.290
Anxious/Depressed	51 (50–58)	62 (51–73)	0.132
Somatic Complaints	52 (50–52)	62 (52–65)	0.075
Withdrawn	53 (53–59)	63 (59–67)	0.071
Externalizing Problems	48 (44–55)	57 (48–69)	0.252
Attention Problems	57 (52–65)	**65** (61–65)	0.413
Aggressive Behavior	50 (50–53)	54 (50–67)	0.388
Sleep problems	51 (51–57)	53 (51–68)	0.288

CBCL-1.5–5 “borderline clinical range” are listed in bold.

**Table III:** Comparison of *T-*scores with normal or borderline/clinical range in CBCL-1.5–5

*T-*scores	All scales normal range (N = 11)	Either scale borderline/clinical range (N = 17) Either scale borderline range (n = 10) Either scale clinical range (n = 7)		

		Normal range n (%)	Borderline clinical range n (%)	Clinical Range n (%)
Total Problems		9 (53)	3 (18)	5 (29)
Internalizing problems		10 (58)	3 (18)	4 (24)
Emotionally Reactive		15 (88)	1 ( 6)	1 ( 6)
Anxious/Depressed		11 (64)	4 (24)	2 (12)
Somatic Complaints		14 (82)	2 (12)	1 ( 6)
Withdrawn		12 (70)	1 ( 6)	4 (24)
Externalizing Problems		11 (64)	3 (18)	3 (18)
Attention Problems		6 (35)	9 (53)	2 (12)
Aggressive Behavior		15 (88)	1 ( 6)	1 ( 6)
Sleep problems		13 (76)	3 (18)	1 ( 6)

*T-*scores	Median (IQR)	Median (IQR)	*P*-value
Total Problems	46 (42–48)	59 (53–64)	0.283
Internalizing problems	41 (41–53)	59 (54–64)	0.178
Emotionally Reactive	50 (50–56)	51 (50–58)	0.705
Anxious/Depressed	50 (50–54)	58 (51–68)	0.115
Somatic Complaints	50 (50–52)	53 (51–62)	0.290
Withdrawn	53 (50–53)	63 (53–68.5)	0.121
Externalizing Problems	44 (40–48)	56 (51–60.5)	0.375
Attention Problems	52 (50–57)	**65** (61–65)	0.020*
Aggressive Behavior	50 (50–51)	52 (50–59)	0.507
Sleep problems	51 (51–51)	53 (51–63)	0.263

* : *P <* 0.05.

CBCL-1.5–5 “borderline clinical range” are listed in bold.

**Table IV:** M-CHAT items associated with the *T*-scores of CBCL-1.5–5 scales (*P*-value)

M-CHAT Items		*T*-scores of CBCL-1.5–5 Scales					

		Internalizing problems	Anxious/Depressed	Somatic complaints	Withdrawn	Attention Problems	Sleep problems
10.	Does not look you in the eye^a^	0.427	0.427	0.024*	0.924	0.825	0.095
12.	Does not smile in response to smile^a^	0.126	0.309	0.764	0.045*	0.283	0.723
**14.**	**Does not respond to name** a	0.328	0.043*	0.862	0.746	0.823	0.028*
**15.**	**Does not look at toy when you point to it** b	0.044*	0.052	0.042*	0.158	0.487	0.098
17.	Does not look at things you look at^a^	0.435	0.405	0.321	0.568	0.027*	0.031*
20.	Have you ever wondered if your child is deaf^b^	0.444	0.052	0.023*	0.916	0.436	0.073
21.	Does not understand what people say^b^	0.051	0.039*	0.206	0.159	0.428	0.813

M-CHAT “critical items” are listed in bold.

* : *P* < 0.05,

a : Mann-Whitney *U* test,

b : Kruskal-Wallis test.
